# Mutagenesis of the odorant receptor co-receptor (*Orco*) reveals severe olfactory defects in the crop pest moth *Helicoverpa armigera*

**DOI:** 10.1186/s12915-022-01411-2

**Published:** 2022-09-30

**Authors:** Xiao-Bin Fan, Bao-Tong Mo, Guo-Cheng Li, Ling-Qiao Huang, Hao Guo, Xin-Lin Gong, Chen-Zhu Wang

**Affiliations:** 1 State Key Laboratory of Integrated Management of Pest Insects and Rodents, Institute of Zoology, Chinese Academy of Sciences, 1 Beichen West Road, Chaoyang District, Beijing, 100101 People’s Republic of China; 2 CAS Center for Excellence in Biotic Interactions, University of Chinese Academy of Sciences, Beijing, People’s Republic of China

**Keywords:** Olfaction, Odorant receptor co-receptor (Orco), CRISPR/Cas9, *Helicoverpa armigera*, EAG, SSR, Chemotaxis

## Abstract

**Background:**

Odorant receptors (ORs) as odorant-gated ion channels play a crucial role in insect olfaction. They are formed by a heteromultimeric complex of the odorant receptor co-receptor (Orco) and a ligand-selective Or. Other types of olfactory receptor proteins, such as ionotropic receptors (IRs) and some gustatory receptors (GRs), are also involved in the olfactory system of insects. Orco as an obligatory subunit of ORs is highly conserved, providing an opportunity to systematically evaluate OR-dependent olfactory responses.

**Results:**

Herein, we successfully established a homozygous mutant (*Orco*^−/−^) of *Helicoverpa armigera*, a notorious crop pest, using the CRISPR/Cas9 gene editing technique. We then compared the olfactory response characteristics of wild type (WT) and *Orco*^−/−^ adults and larvae. *Orco*^−/−^ males were infertile, while *Orco*^−/−^ females were fertile. The lifespan of *Orco*^−/−^ females was longer than that of WT females. The expressions of most *Ors*, *Irs*, and other olfaction-related genes in adult antennae of *Orco*^−/−^ moths were not obviously affected, but some of them were up- or down-regulated. In addition, there was no change in the neuroanatomical phenotype of *Orco*^−/−^ moths at the level of the antennal lobe (including the macroglomerular complex region of the male). Using EAG and SSR techniques, we discovered that electrophysiological responses of *Orco*^−/−^ moths to sex pheromone components and many host plant odorants were absent. The upwind flight behaviors toward sex pheromones of *Orco*^−/−^ males were severely reduced in a wind tunnel experiment. The oviposition selectivity of *Orco*^−/−^ females to the host plant (green pepper) has completely disappeared, and the chemotaxis toward green pepper was also lost in *Orco*^−/−^ larvae.

**Conclusions:**

Our study indicates that OR-mediated olfaction is essential for pheromone communication, oviposition selection, and larval chemotaxis of *H. armigera*, suggesting a strategy in which mate searching and host-seeking behaviors of moth pests could be disrupted by inhibiting or silencing *Orco* expression.

**Supplementary Information:**

The online version contains supplementary material available at 10.1186/s12915-022-01411-2.

## Background

Insects live in an ever-changing chemical world comprising a variety of odors [1]. Olfaction plays a significant role in the insect life cycle and regulates many important behaviors, such as host plant location [2], mate choice [3], oviposition site selection [4], predator avoidance [5], and behavioral division [6]. Insect olfaction has, thus, attracted considerable research attention.

Insects have an extremely complex olfactory system, which is responsible for the detection of different types of odors. The antennae and maxillary palps, two major olfactory organs in insects, are covered with different types of olfactory sensilla (trichoid, basiconic, and coeloconica sensilla), which contain olfactory sensory neurons (OSNs) [7, 8]. The antennal lobe (AL), which contains many globule-shaped neuropils known as glomeruli, is the primary olfactory processing center responsible for the integration of peripheral olfactory information [9]. Odorant receptors (ORs) expressed in the dendrite membranes of OSNs are important in the insect olfactory signal transduction pathway and have been studied in depth [10]. ORs are sensitive to many different types of compounds, including insect pheromone components [11] and host plant compounds, such as esters, alcohols, and ketones [12, 13]. Other types of chemosensory receptors, such as ionotropic receptors (IRs) and gustatory receptors (GRs), have also been identified in the insect peripheral olfactory system [14]. IRs are mainly for responding to volatile amines and acids [12, 13, 15, 16]. For example, in *Drosophila melanogaster*, DmIr92a is involved in sensing amines [17], and DmIr8a and DmIr64b are required for response to carboxylic acids (such as acetic acid and HCl) [16]. In addition, 2–3 GRs are involved in the detection of CO_2_ in insects [18, 19]. DmGr21a and DmGr63a are involved in the rejection of CO_2_ in *Drosophila* [20]. Using these different types of chemosensory receptors, insects can complete important behaviors such as mating, oviposition, and host selection in their natural habitats.

Insect ORs are odorant-gated ion channels that are formed by a heteromultimeric complex of the odorant receptor co-receptor (Orco) and a tuning Or [21–27]. Both Orco and Ors possess seven inverted transmembrane domains (TMDs), with an intracellular N-terminus and an extracellular C-terminus, which is opposite to the topology of vertebrate olfactory receptors that are conventional G-protein coupled receptors (GPCRs) [25]. The number of Ors varies greatly among insect species. For example, up to 400 CfloOrs are expressed in the social ant *Camponotus floridanus* [28], while only 10 PhumOrs are expressed in *Pediculus humanus* [29]. The sequence similarity of Ors is very low (the highest similarity is only 20%), whereas Orco (as an obligate partner) is highly conserved among insect species, especially toward the C terminal, and there is a single *Orco* gene in each insect species [30, 31].

Studies of ORs have mainly focused on the characterization of tuning profiles of each Or in *Xenopus oocytes* [32] and *Drosophila* OSNs [33]. Some Ors are narrowly tuned, while others are broadly tuned to diverse odorants and coding is combinatorial [34]. Pheromone receptors (PRs) are a subset of Ors responsible for olfactory detection of sex pheromone compounds. In general, the tuning spectrum of PRs is specific [35, 36]. For example, BmOr1 and BmOr3 are specifically tuned to bombykol and bombykal, respectively, in *Bombyx mori* [37, 38]. Conventional Ors are usually tuned to host plant volatiles and help insects to locate and select host plants. In *B. mori*, BmOr56 is specifically tuned to the compound *cis*-jasmone emitted by mulberry leaves, which strongly attracts silkworm larvae [39]. Two PxylOrs (PxylOr35 and PxylOr49) in *Plutella xylostella* are specifically tuned to isothiocyanates in cruciferous plants, guiding the adults to these plants [40]. HassOr31 is highly expressed in the ovipositor of *Helicoverpa assulta* and widely tuned to 12 plant odorants including Z-3-hexenyl butyrate, acting as a key chemical cue for locating oviposition sites [41]. However, in *Manduca sexta*, plant-seeking and oviposition behaviors of the *Orco* knocking-out adults were sustained although foraging and pollination behaviors were disrupted [42]. This proves that the OR-mediated olfactory responses are not the whole olfactory responses of insects. Orco, as an obligate partner, is essential for each functional Or. When *Orco* is knocked out, the Or functional repertoire will be abolished. Thus, *Orco* is a prime candidate to study OR-dependent olfactory responses in the insect olfactory system. It has been showed that the Orco family can form functional ion channels in the absence of a tuning Or, and its agonist VUAA1 is capable of gating orthologues across multiple insect taxa [43].

As a typical polyphagous insect, the cotton bollworm, *Helicoverpa armigera*, is an important agricultural pest globally. A total of 84 candidate OR genes have been identified in the published genomic data of *H. armigera* [44] and at least 65 candidate OR transcripts (1 *Orco*, 7 *PRs*, at least 55 conventional *Ors*), and at least 21 *Irs* have been identified in the transcriptome data of *H. armigera* [45–49]. Seven PRs are involved in sex pheromone communication between male and female *H. armigera*, which are mainly expressed in three types of trichoid sensilla in male antennae. Among them, type A sensilla respond to the sex pheromone component Z11-16: Ald, type B sensilla to the behavior antagonist Z9-14: Ald, and type C sensilla to another sex pheromone component Z9-16: Ald and the behavior antagonists Z9-14: Ald and Z9-16: OH [50]. To date, the functions of most PRs have been studied. HarmOr13 is tuned to Z11-16: Ald [51, 52], HarmOr6 to Z9-16: Ald and Z9-14: Ald [51], and Z9-16: OH [47, 53], HarmOr14b to Z9-14: Ald [11, 47, 52], and HarmOr16 to Z9-14: Ald and Z11-16: OH [52]. The functions of 29 conventional Ors have been identified, and they are mainly responsible for detecting the green leaf volatiles, terpenes, aliphatic, and aromatic compounds of plants involved in searching for nectar and the selection of host plants in *H. armigera* [54, 55]. Most Ors have a broad tuning spectrum. For example, HarmOr60 expressed in larval antennae can respond to 25 different compounds, with cis-3-hexen-ol-1 being the most effective ligand. Some Ors are narrowly tuned, such as HarmOr42 expressed in adult antennae, which is specifically tuned to phenylacetaldehyde [54, 55]. However, the functions of more than half of the Or repertoire in *H. armigera* are still unknown, and the role of the OR-mediated olfactory pathway in the olfactory system is still not fully understood. In recent years, the CRISPR/Cas9 system has been used for functional studies of HarmOr14 [3], HarmOr16 [56], HarmOr42 [55], PBP1 [57], and SNMP1 [58] of *H.* *armigera*. In this study, we employed CRISPR/Cas9 gene editing to generate the *Orco* mutant of *H. armigera*. Next, we compared the antennal transcriptional profiles, electrophysiology, and neuroanatomical phenotypes of the mutant and WT adults. Moreover, we examined the behaviors of their adults and larvae. Taken together, our results reveal the OR-mediated olfaction play an essential role in mate searching and host plant selection of *H.* *armigera*.

## Results

### Generation of the *Orco* homozygous mutant of *H. armigera*

**Fig. 1:**
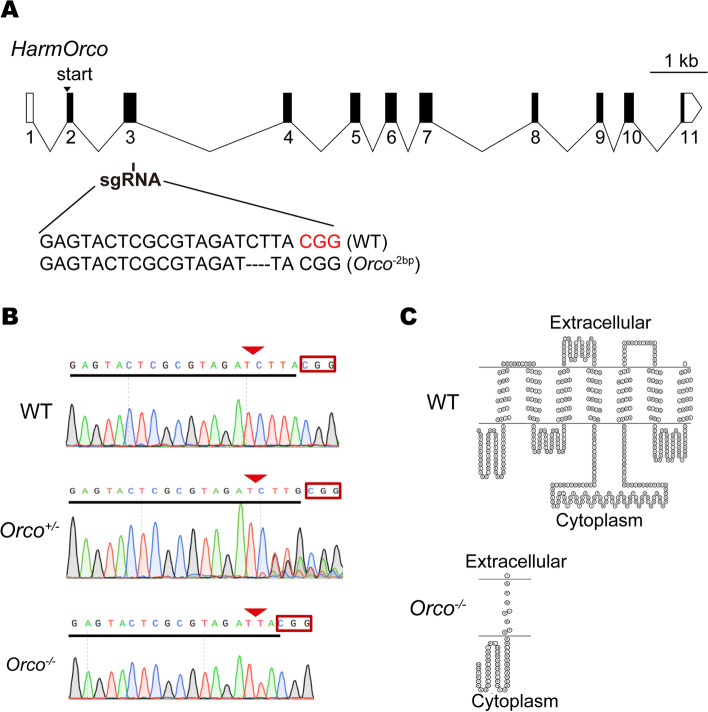
CRISPR/Cas9 directed heritable mutagenesis of *Orco* in *Helicoverpa armigera.* **A** Schematic of the *Orco* structure. Exons are shown as boxes and introns as bent lines. Shaded boxes represent the coding sequences of the *Orco* gene. An sgRNA targeting the sequence of exon 3 is indicated (PAM: red). A representation of mutations with a 2-bp deletion (− 2 bp) is shown (*Orco*^−2 bp^). **B** DNA sequencing chromatograms of *Orco* in the wild type (WT) (top), heterozygous mutant (*Orco*^+*/−*^) (middle), and homozygous mutant (*Orco*^−/−^) (bottom). Targeted sequences are underlined and PAM sequences (CGG) are in boxes (inverted triangles indicate the expected cleavage sites of the Cas9 protein). **C** Predicted secondary structure of Orco in the WT (top) and Orco in the homozygous mutant (*Orco*^−/−^) (bottom). These models were predicted using the software TOPO2 (http://www.sacs.ucsf.edu/TOPO2/) based on the methods [11]

**Fig. 2:**
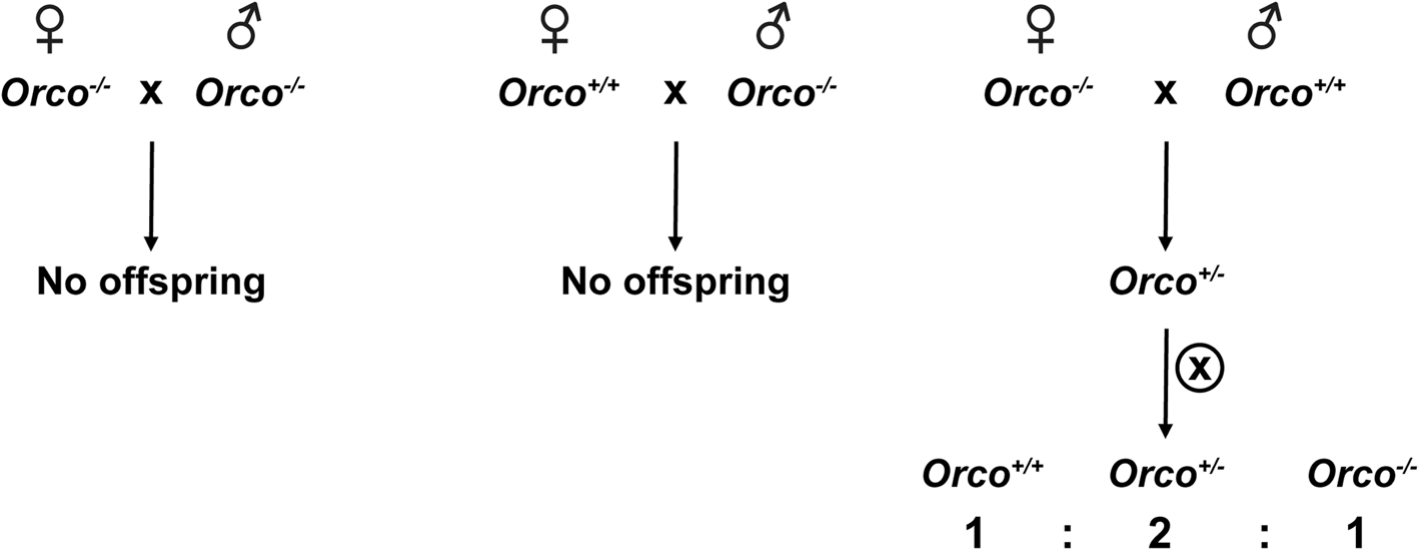
Three different hybrid combinations of homozygous individuals (*Orco*^−/−^) and wild type *Helicoverpa armigera* individuals. In each group, the individual on the left is female and the individual on the right is male. The third group was also used to maintain the homozygous mutant (*Orco*^−/−^) in each generation

To obtain *Orco* mutant lines of *H. armigera*, we employed the CRISPR/Cas9 gene editing system, which has been successfully developed in many different organisms [59–61]. A single-guide RNA (sgRNA) targeting the third exon of the *Orco* (Fig. 1A) was designed using the tool CHOPCHOP [62]. A mixture of sgRNA (200 ng/µL) and Cas9 protein (100 ng/µL) was co-injected into nearly 711 embryos. Then 77 injected embryos were successfully hatched, and finally, 48 G0 adults were obtained. To screen for targeted mutations, genomic DNA was extracted from one of the hind legs of each G0 adult, then confirmed by both PCR and DNA sequencing. Among 48 G0 adults, 36 G0 harbored multiple targeted mutations (Table 1), indicating a high mutation efficiency (75%) in G0 adults. Each mutated G0 was mated with three wild type (WT) heterosexual adults to produce G1 offspring. From the 36 crosses, 13 (36.1%) G0 adults produced heterozygous mutant progeny in G1, suggesting that G0 adults carried stable and heritable mutagenesis (Table 1, Additional file 1: Figure S1A). One of the *Orco* mutations with a 2-bp deletion at exon 3 was inferred to produce a nonfunctional, truncated Orco protein and was selected for subsequent crosses (Fig. 1B, C). Heterozygous moths (*Orco*^+/−^) in G1 were self-crossed with their siblings and homozygous mutant moths (*Orco*^−/−^) were obtained in the G2 generation (Fig. 1B). Interestingly, no offspring were produced when homozygous mutant males were mated with homozygous mutant females or WT females but fertile offspring were produced from homozygous mutant females mated with WT males (Fig. 2). Hence, in order to obtain sufficient *Orco*^−/−^ for the experiments, *Orco*^+/−^ were maintained for self-crossing to screen *Orco*^−/−^ in subsequent generations (Fig. 2). There were no distinct morphological differences between *Orco*^−/−^ and WT adults (Additional file 1: Figure S1B). Therefore, each adult used was genotyped by sequencing prior to the experiments.

**Table 1:** Summary of the CRISPR/Cas9 directed mutations from G0 to G2

		Total number	Mutated number	Germline mutation
G0	Injected embryo	711	/	/
	Hatched larvae	77	/	/
	Adults	48	36 (75%)	13 (36.1%)
		Wild type	Heterozygotes	Homozygous
G1	Adult	49	21 (− 2 bp)	0
		5	9 (− 5 bp)	0
		24	17 (− 1, + 4 bp)	0
		51	8 (− 7 bp)	0
		20	17 (+ 3 bp)	0
		34	41 (− 12 bp)	0
		23	36 (− 2, + 7 bp)	0
		12	7 (− 4 bp)	0
		9	8 (− 1, + 5 bp)	0
		21	19 (− 1 bp)	0
		7	10 (− 1, + 3 bp)	0
		11	13 (− 3 bp)	0
		33	21 (− 8 bp)	0
	Total	299 (56.84%)	227 (43.15%)	0
		Wild type	Heterozygotes	Homozygous (− 2 bp)
G2	Adults	27 (24.54%)	48 (43.63%)	35 (31.82%)

**Fig. 3:**
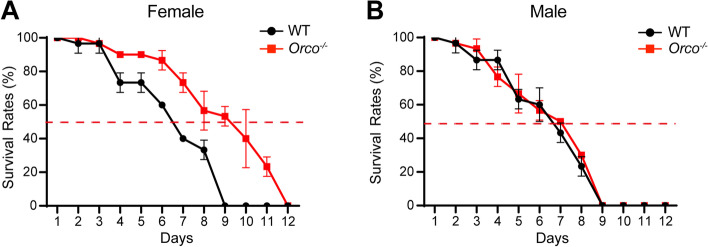
Comparisons of lifespans between wild type (WT) and *Orco*^−*/*−^ adults of *Helicoverpa armigera*: **A** female, **B** male. The *x*-axis shows days after eclosion, and the *y*-axis indicates the survival rate. Thirty WT and mutant adults that emerged on the same day were used for survival rate observations. Error bars, mean ± SD

It has been reported that olfaction could regulate the longevity of *Drosophila* and ants [63, 64]. Thus, we compared the lifespans of *Orco*^−/−^ and WT adults (Fig. 3A, B). *Orco*^−/−^ females exhibited a 48% increase in median life span compared with WT females (Fig. 3A), but the lifespan of *Orco*^−/−^ males was indistinguishable from that of WT males (Fig. 3B).

### Antennal transcriptome sequencing of *Orco*^*−/*−^ and WT adults

**Fig. 4:**
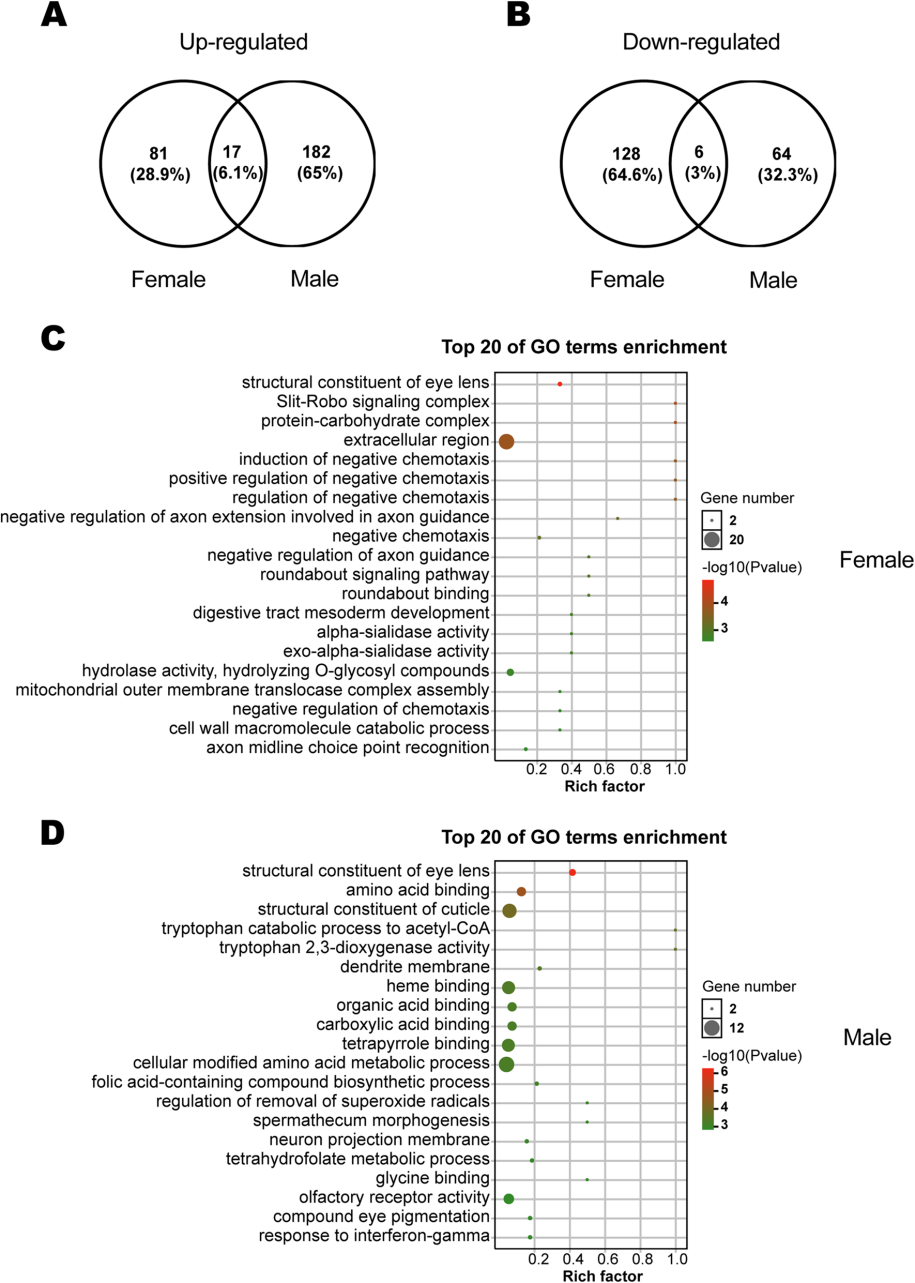
Transcriptional profiling revealed changes in gene expression after the gene *Orco* was knocked out in adult *Helicoverpa armigera* antennae. **A** Up-regulated and **B** down-regulated differentially expressed genes (DEGs) after *Orco* was knocked out in females and males. The top 20 gene ontology terms with significant enrichment in females (**C**) and males (**D**)

In order to detect transcriptional changes induced by the loss of Orco, antennal transcriptome sequencing of *Orco*^−/−^ and WT adults was performed. Differentially expressed genes (DEGs) were identified with the thresholds (a log_2_ fold change > 1 and *P* value < 0.05) (Fig. 4A, B). The results clearly showed that, compared with genes from WT adults, the numbers of upregulated genes in the antennae of female and *Orco*^−/−^ males were 98 and 199, respectively, and the numbers of downregulated genes were 134 and 70 (Fig. 4A, B). Gene ontology (GO) enrichment analyses were conducted for males and females. Among the 20 significantly enriched pathways in *Orco*^−/−^ females, there were five related to negative chemotaxis (“induction of negative chemotaxis,” “positive regulation of negative chemotaxis,” “regulation of negative chemotaxis,” “negative chemotaxis,” and “negative regulation of chemotaxis”) and three related to axon orientation (“negative regulation of axon guidance,” “negative regulation of axon extension involved in axon guidance,” and “axon midline choice point recognition”) (Fig. 4C). While in *Orco*^−/−^ males, there were two allelopathy-related pathways (“olfactory receptor activity” and “neuron projection membrane”) (Fig. 4D).

**Fig. 5:**
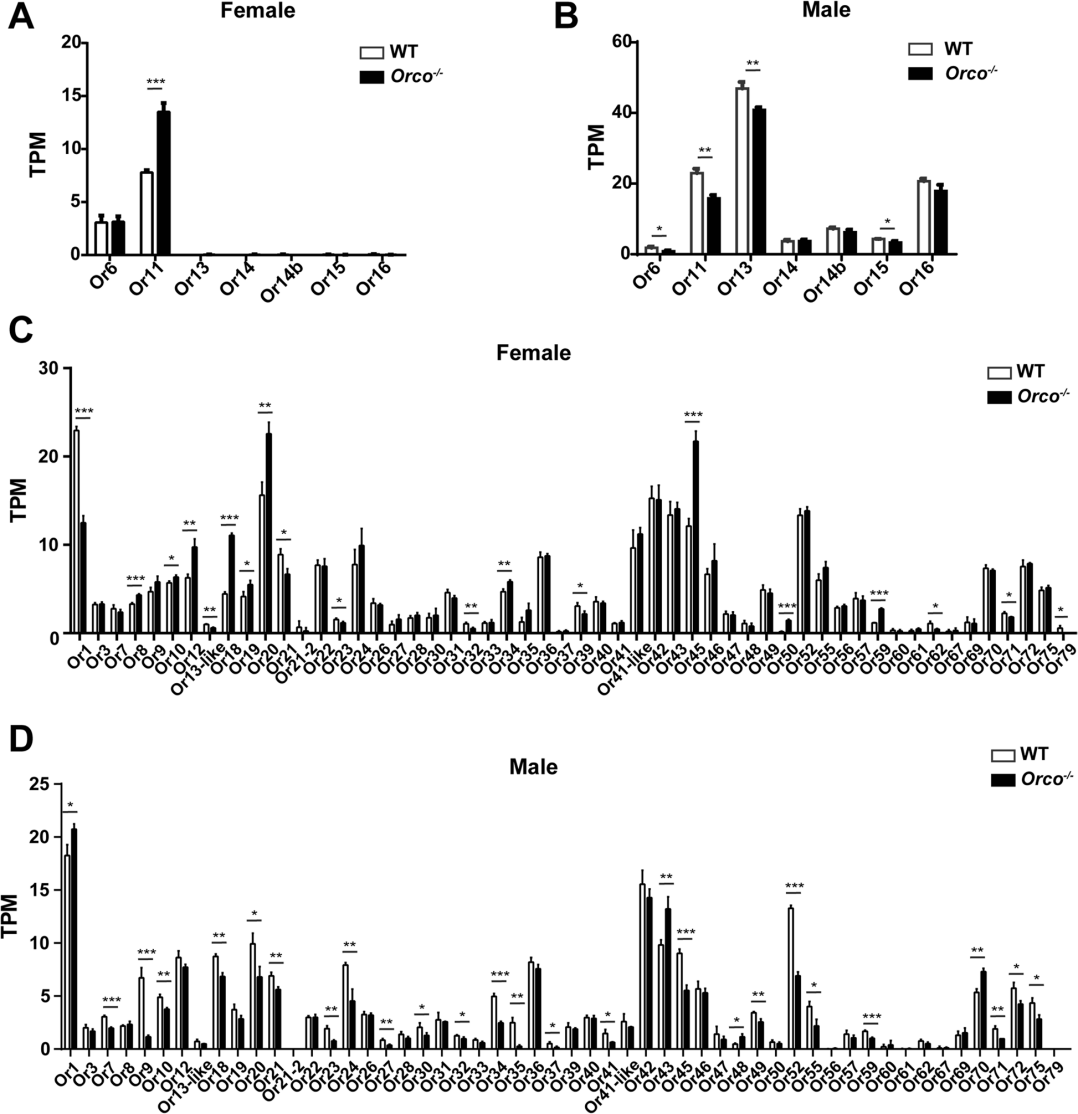
The TPM value (transcripts per million) of the main *Ors* in *Orco*^−/−^ adults and WT *Helicoverpa armigera* through RNA-seq. **A, B** TPM of pheromone receptors (*PR*s) in females and males. **C, D** TPM of conventional *Or*s in females and males. Multiple *t*-test was used: *, *P* < 0.05; **, *P* < 0.01; ***, *P* < 0.001. Error bars, mean ± SD

We also compared the expression of 61 *Or*s (including 7 *PR*s and 54 conventional *Or*s), 27 *Ir*s, and 9 other olfaction-related genes (including *Gr1*, *Gr2*, *Gr3*, *TRPA1*, *PBP1*, *PBP2*, *PBP3*, *GOBP1*, and *GOBP2*) between the antennae of *Orco*^−*/*−^ and WT adults (Additional file 2: Table S1). For the seven PRs, only *Or11* and *Or6* were highly expressed in WT females. The expression level of *Or11* in the antennae of *Orco*^−*/*−^ females was 73.41% higher than that in WT females and there was no significant difference in the expression level of *Or6* (Fig. 5A). The expression levels of *Or6*, *Or11*, *Or13*, and *Or15* in the antennae of *Orco*^−*/*−^ males were 51.55%, 31.27%, 12.87%, and 22.70% lower than those in the WT males, respectively, and the expression levels of *Or14*, *Or14b*, and *Or16* were not significantly different (Fig. 5B).

For the 54 conventional *Or*s, the expression levels of 10 *Or*s were higher in the antennae of *Orco*^−*/*−^ females than those in the WT, the expression levels of 9 *Or*s were lower, and there were no significant differences in the expression levels of the other 35 *Or*s (Fig. 5C). In *Orco*^−*/*−^males, the expression levels of 4 *Or*s were higher than those of the WT males, the expression levels of 23 *Or*s were lower, and the expression levels of the other 27 *Or*s were not changed (Fig. 5D).

For the 27 identified *Ir*s, the expression level of one *Ir* in the antennae of *Orco*^−*/*−^ females was higher than that in the WT females. The expression levels of 6 *Ir*s were lower in the antennae of *Orco*^−*/*−^ females, and the expression levels of the other 20 *Ir*s were not significantly different (Additional file 3: Figure S2A). Compared with WT males, *Orco*^−*/*−^ males had 5 *Ir*s with higher expression levels, 6 *Ir*s with lower expression levels, and 16 *Ir*s with no difference in expression levels (Additional file 3: Figure S2B).

In addition, we also detected the expression levels of nine olfaction-related genes (*Gr1*, *Gr2*, *Gr3*, *TRPA1*, *PBP1*, *PBP2*, *PBP3*, *GOBP1*, and *GOBP2*). In *Orco*^−*/*−^females, both *Gr1* and *Gr2* showed lower expression levels than in WT females; *TRPA1*, *PBP2*, *PBP3*, *GOBP1*, and *GOBP2* showed higher expression levels than in WT females, while the expression levels of *Gr3* and *PBP1* did not differ (Additional file 3: Figure S2C, D). In *Orco*^−*/*−^ males, four genes (*Gr1*, *PBP2*, *PBP3*, and *GOBP1*) had lower expression levels than in WT males. One gene (*PBP1*) showed higher expression levels in WT than mutant females but the expression levels of other genes (*Gr2*, *Gr3*, *TRPA1*, and *GOBP2*) did not differ (Additional file 3: Figure S2E, F).

In sum, the expression levels of some *PR*s in males were affected, while the expression levels of most *Or*s, *Ir*s, and other olfaction-related genes were not affected when *Orco* was knocked out.

### Morphology of the antennal lobes in *Orco*^−/−^ and WT adults

**Fig. 6:**
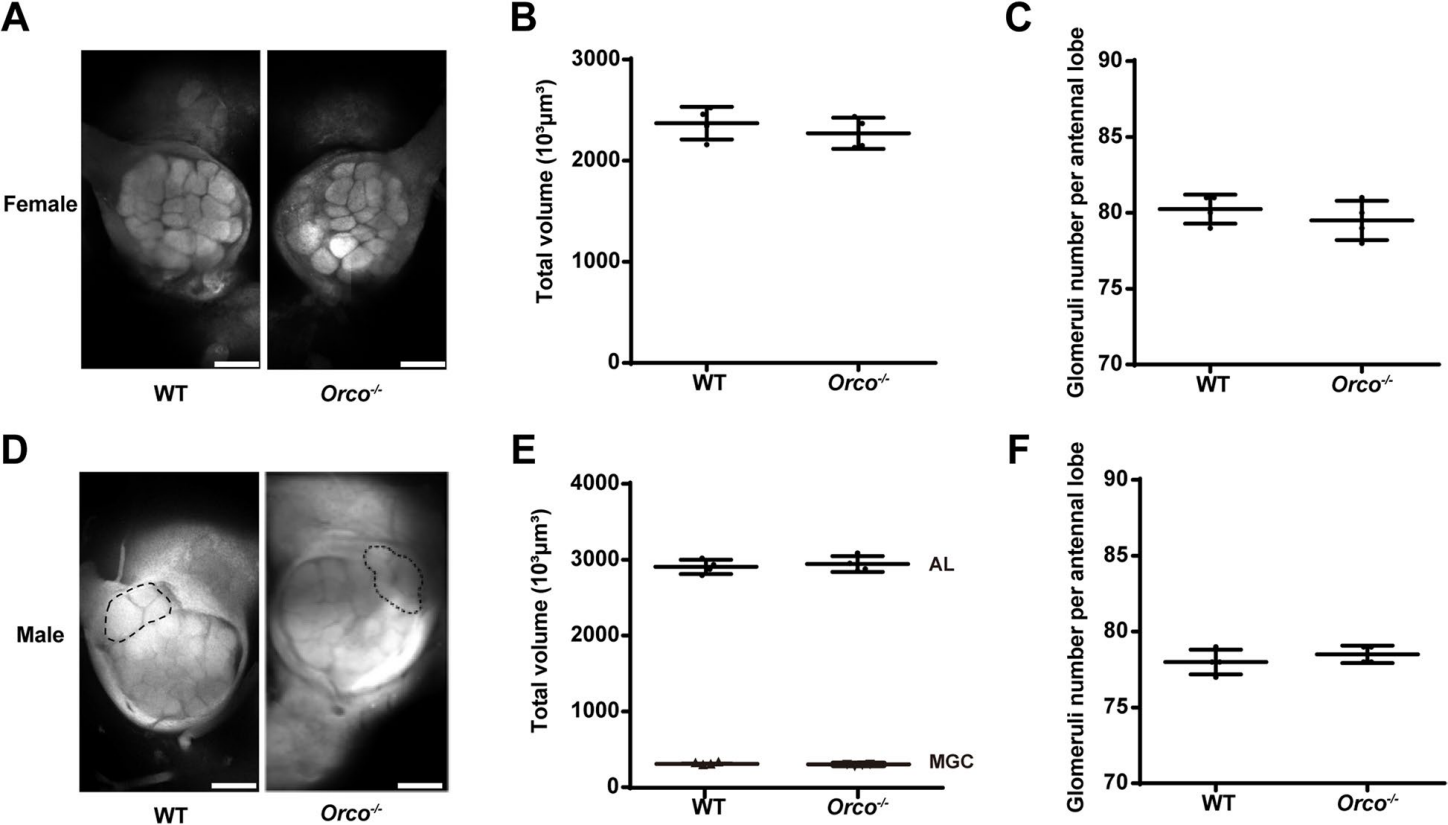
Neuroanatomical phenotypes of wild type (WT) and *Orco*^−*/*−^ *Helicoverpa armigera*. **A** Confocal images of the female WT and mutant antennal lobe (AL). **B** Volume of all glomeruli from WT (*n* = 4) and mutant female (*n* = 4) ALs. **C** Number of glomeruli in the ALs of WT (*n* = 4) and mutant females (*n* = 4). **D** Confocal images of the ALs of WT and mutant males. Areas of the macroglomerular complex (MGC) in the ALs are indicated by dotted lines. **E** Volume of all glomeruli and volume of the MGC area in ALs of WT (*n* = 4) and mutant males (*n* = 4). **F** Number of glomeruli in the ALs of WT (*n* = 4) and mutant males (*n* = 4). Confocal images were taken using 20 × 0.6 objective. Scale bars = 100 µm. Error bars, mean ± SD

To detect if there were changes in the neuroanatomical phenotype of the antennal lobe (AL) in *H. armigera Orco*^−*/*−^ adults, we compared the total volume and total number of glomeruli in the AL of *Orco*^−*/*−^ and WT adults (Fig. 6). In both females and males, the total volume and total number of glomeruli in a single AL of *Orco*^−*/*−^ adults did not differ from those of WT adults. The volume and number of macroglomerular complex (MGC) regions also showed no difference (Fig. 6).

### EAG responses to pheromone compounds and other odorants of *Orco*^−/−^ and WT adults

**Fig. 7:**
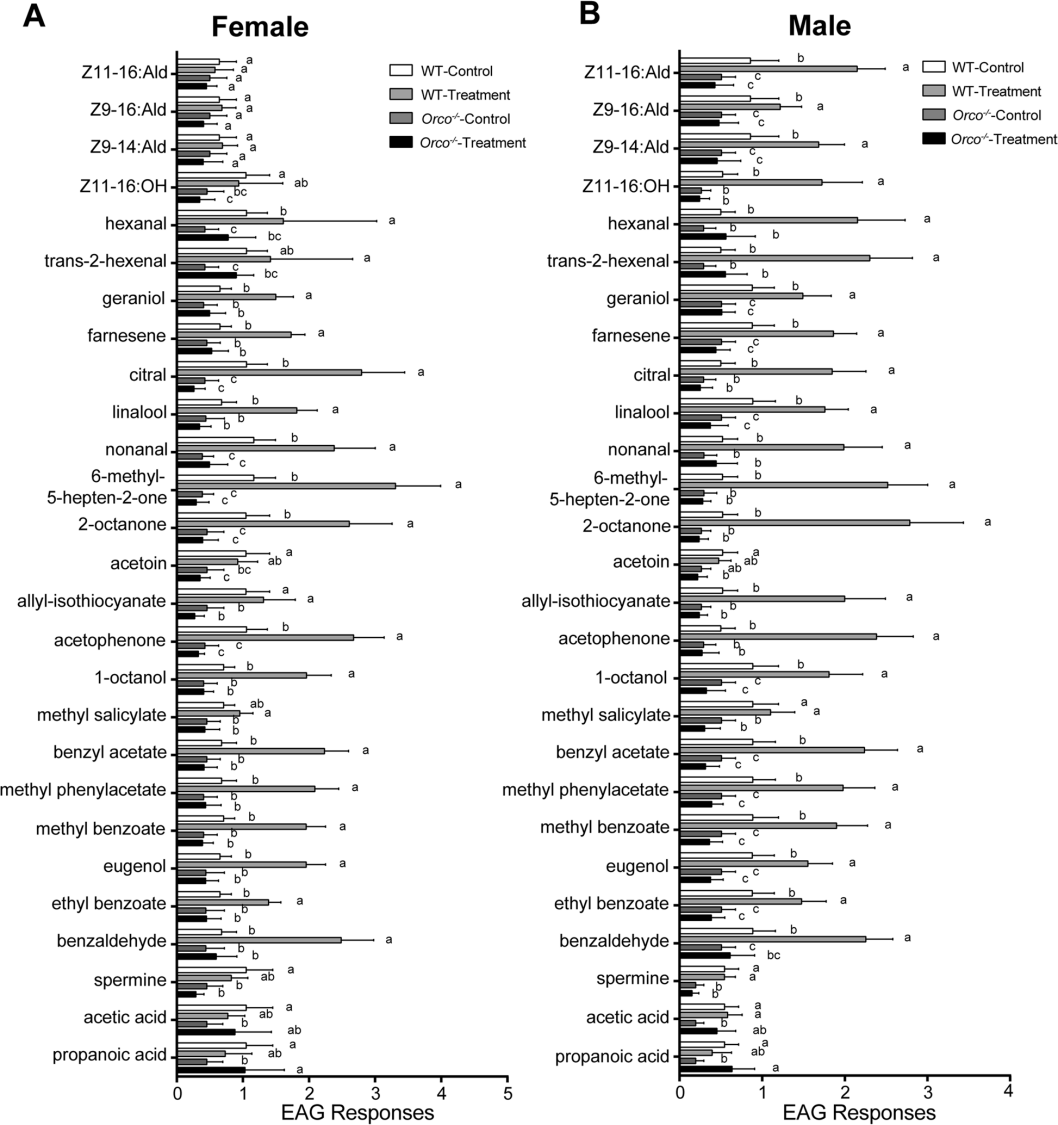
Comparisons of antennal EAG responses to a series of odors in wild type (WT) and *Orco*^−*/*−^ *Helicoverpa armigera* adults: **A** female; **B** male. For comparisons in each group, six or ten WT and mutant individuals were used, respectively. Significant differences (ANOVA followed by Tukey’s test; *P* < 0.05) are marked by different letters. Error bars, mean ± SD

To evaluate the role of *Orco* in the olfactory pathway, antennal EAG analysis was conducted on *Orco*^−/−^ and WT adults. In total, 27 different types of chemical compounds were used for EAG analysis (Fig. 7; Additional file 4: Table S2). For female moths, our results clearly showed that WT females had robust antennal EAG responses to 20 plant odorants; however, limited responses to these compounds were observed in *Orco*^−/−^ females (Fig. 7A). For male moths, strong antennal EAG responses to 23 compounds (including four sex pheromone-related components) were observed in WT males, while *Orco*^−/−^ males were anosmic to these plant odorants and sex pheromone-related components (Fig. 7B). Furthermore, we found that both sexes of *Orco*^−/−^ and WT showed no significant response to propanoic acid, acetic acid, and spermine.

### Electrophysiological responses of trichoid sensilla to pheromone-related compounds in *Orco*^−/−^ and WT males

**Fig. 8:**
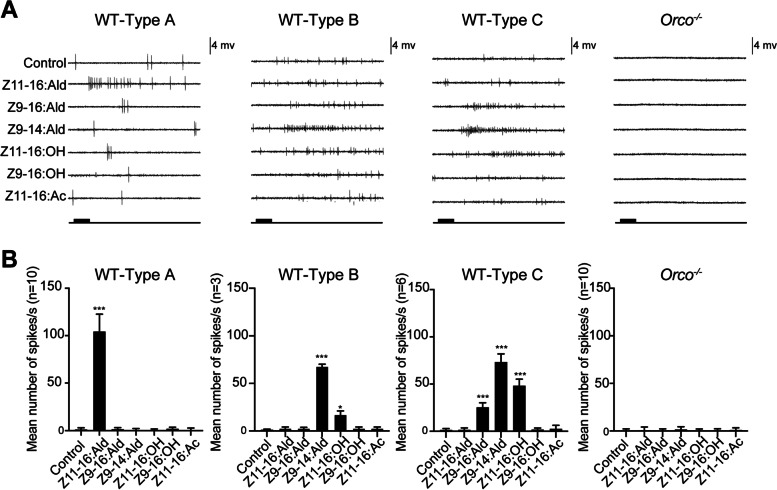
Comparison of antennal responses to the main sex pheromone components in wild type (WT) and *Orco*^−*/*−^ *Helicoverpa armigera* adults, assessed using single sensillum recordings (SSRs). **A** Representative response profiles of WT-type A, WT-type B, WT-type C, and *Orco*^−*/*−^. **B** Spike frequencies represented with histograms of WT-type A, WT-type B, WT-type C, and *Orco*^−*/*−^. The number of spikes was counted from the first 500 ms of the response. Paraffin oil was used as a control (multiple *t*-test: *, *P* < 0.05; **, *P* < 0.01; ***, *P* < 0.001). Error bars, mean ± SD

Single sensillum recordings (SSRs) were further performed to compare antennal sensilla responses to sex pheromone compounds between WT and *Orco*^−/−^ males. Following the method reported for mosquitoes [4], we processed the SSR assay with a random sampling of antennal sensilla from WT and *Orco*^−/−^ males. In total, responses were recorded from 72 and 70 antennal sensilla from WT and *Orco*^−/−^ males, respectively. We readily located and detected three types of sensilla in WT males: 72.22% type A sensilla responded to Z11-16: Ald, 4.17% type B sensilla responded to Z9-14: Ald, 8.33% type C sensilla responded to Z9-16: Ald and Z9-14: Ald. In contrast, none of the recorded antennal sensilla responded to these sex pheromone components in *Orco*^−/−^ males (Fig. 8, Additional file 5: Figure S3).

### Behavioral responses to sex pheromones in *Orco*^−/−^ and WT males

**Fig. 9:**
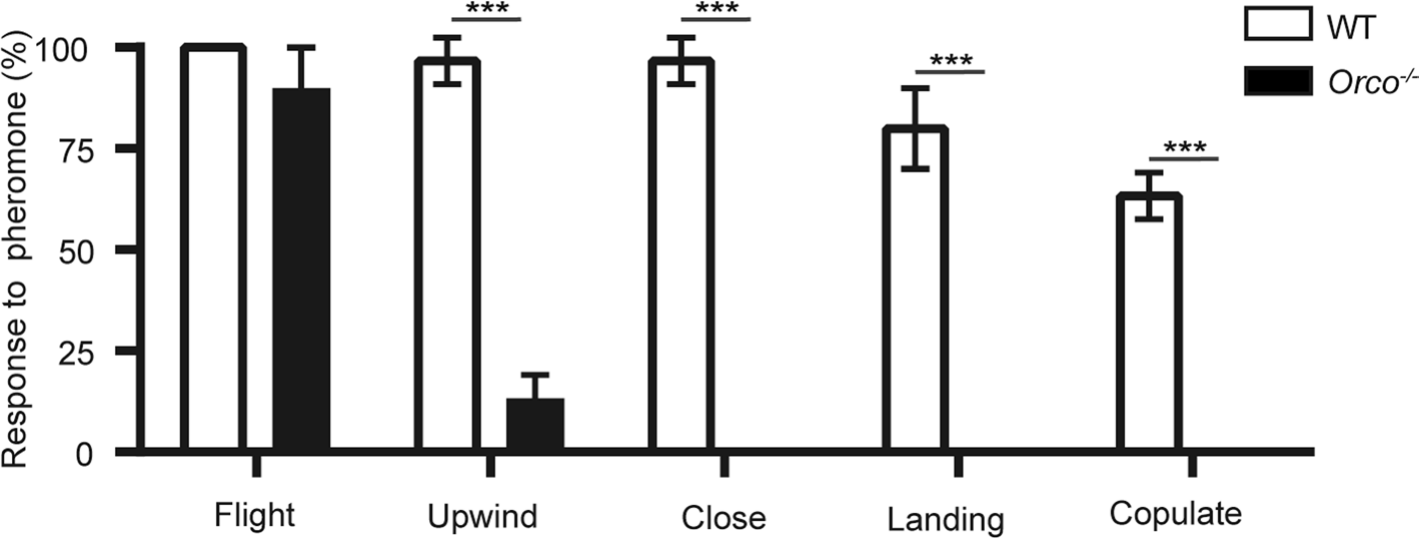
Behavioral responses of wild type (WT) and *Orco*^−*/*−^ *Helicoverpa armigera* males to the sex pheromone blend (Z11-16: Ald + Z9-16: Ald, 97:3) in the wind tunnel. Shaded box: WT males (*n* = 30); blank box: *Orco*^−*/*−^ mutant males (*n* = 30). Multiple *t*-test: *, *P* < 0.05; **, *P* < 0.01; ***, *P* < 0.001. Error bars, mean ± SD

A wind tunnel assay was performed to detect differences in attractiveness to a blend of the main sex pheromones [Z11-16: Ald and Z9-16: Ald (97:3)] between *Orco*^−/−^ and WT males. The WT males showed a robust response to the main pheromone blend, exhibiting male sexual behaviors, including flight, upwind, close, landing, and copulate (Fig. 9, Additional file 6: Video S1). The *Orco*^−/−^ males also took off in the wind tunnel, but only 13% of adults exhibited upwind flight behavior. Specifically, the behaviors of close flight, landing, and copulate were completely absent in mutant males (Fig. 9, Additional file 7: Video S2).

### Oviposition preferences of *Orco*^*−/*−^ and WT females

**Fig. 10:**
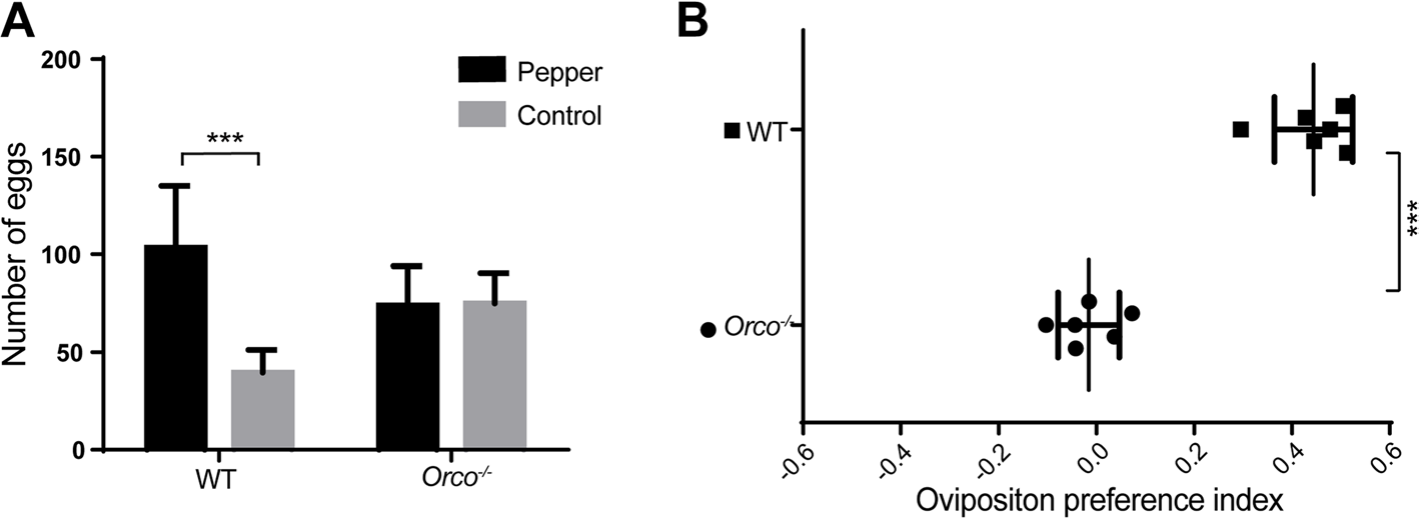
Oviposition preference experiment of wild type (WT) and *Orco*^−*/*−^ *Helicoverpa armigera* females. **A** Number of eggs distributed in area of green pepper fruit discs and control. A multiple *t*-test was used to detect differences between treatments (*, *P* < 0.05; **, *P* < 0.01; ***, *P* < 0.001). **B** Oviposition preference index between the host plant (green pepper) and control. The formula (*T* − *C*)/(*T* + *C*) was used to calculate the preference index, where *T* is the number of eggs in the host plant area, and *C* is the number of eggs in the control area. A multiple *t*-test was used to detect differences between treatments (*, *P* < 0.05; **, *P* < 0.01; ***, *P* < 0.001). Error bars, mean ± SD

A simple dual-choice oviposition assay was performed using *Orco*^−/−^ and WT females (Additional file 8: Figure S4). The mean number of eggs was 2.6 times higher in areas exposed to fresh green pepper fruit discs than in areas with no fruit discs (Fig. 10A). The preference index was 0.44 ± 0.07 (mean ± SD) (Fig. 10B), suggesting a significant preference for oviposition on green pepper fruit discs. When *Orco*^−/−^ females were tested, there was no obvious difference in the numbers of eggs between treatments (Fig. 10A); thus, the preference index was almost zero (Fig. 10B). Our results clearly suggested that *Orco*^−/−^ females lost the oviposition preference for green pepper fruit discs.

### Chemotaxis of *Orco*^−/−^ and WT larvae

**Fig. 11:**
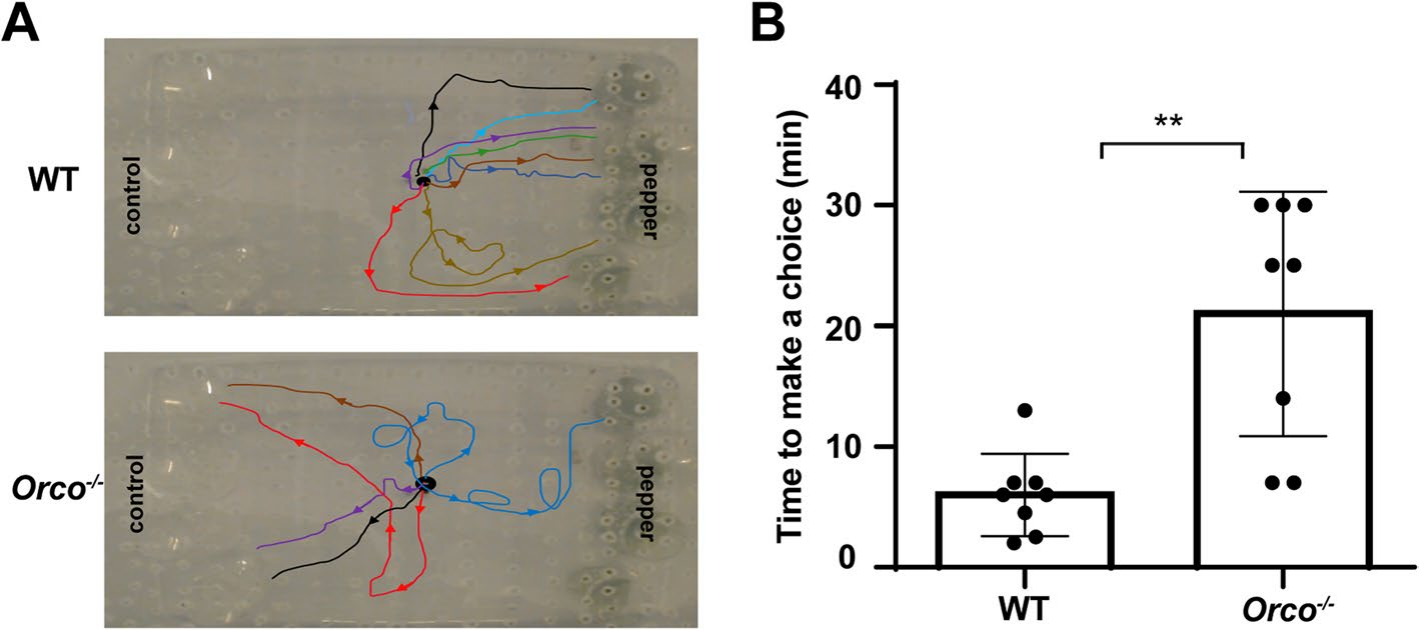
Chemotaxis of wild type (WT) and *Orco*^*−/−*^ *Helicoverpa armigera* larvae. **A** Typical trajectories of WT (upper) and mutant larvae (below). Eight biological replications were conducted for each genotype. In the mutant group, three mutants that remained inert for 30 min were excluded from the trajectory description. **B** The time spent to make a choice in WT and *Orco*^−*/*−^ larvae. Error bars, mean ± SD

A larval chemotaxis test (Additional file 9: Figure S5) was performed to detect whether Orco is necessary for food selection by *H. armigera* larvae. Our results showed that eight WT larvae chose the green pepper (Fig. 11A) and the mean time to make a choice was 6 ± 3 min (mean ± SD) (Fig. 11B). However, only one *Orco*^−*/*−^ larva chose the green pepper, four larvae chose the control, and the other three larvae remained still for 30 min (Fig. 11A). The mean time for choices was longer at nearly 21 ± 9 min (mean ± SD) (Fig. 11B).

## Discussion

Insects sense a variety of volatile molecules using different types of olfactory receptor proteins (Ors, Irs, Grs, and TRPA1). As a notorious worldwide agricultural pest, *H. armigera* uses its complex olfactory system to find mates and nectar and select host plants. In this study, we explored the role of the OR-mediated olfactory pathway in the foraging and reproductive behavior of *H.* *armigera*, using an *Orco* homozygous mutant, established using CRISPR/Cas9, in which OR-mediated olfaction was nonfunctional and demonstrated that OR-mediated olfaction is essential for pheromone communication, oviposition selection, and larval chemotaxis of *H.* *armigera*.

The type II CRISPR/Cas system (only one Cas9 protein required) was used in our study owing to its simplicity and precision [65, 66]. Through direct injection of synthesized sgRNA and Cas9 protein into newly laid embryos, a high mutation efficiency (75%) was obtained in G0 adults, which was similar to efficiencies reported in other insects [60, 67, 68]. After screening in the next generations, a homozygous *Orco* mutant line with a 2-bp deletion was obtained. There were no obvious changes in the appearance of mutant moths, so it was necessary to identify the genotype of each adult prior to the experiments using DNA sequencing of the targeted genomic region by PCR amplification. However, this method was time consuming and labor intensive. In *Anopheles coluzzii* and silkworm *Orco* mutants, this obstacle was overcome by distinguishing mutants using a DsRed visible eye color marker and fluorescent proteins, respectively [4, 69]. In future studies with *H. armigera*, CRISPR-based applications, such as knock-ins and transgenesis with the aid of a *piggyBac* transposon [70], should be explored.

Although there were no apparent morphological differences between the *Orco*^−*/*−^ and WT adults, no offspring were produced when *Orco*^−*/*−^ males were mated with *Orco*^−*/*−^ females or WT females (Fig. 2). Thus, we could not maintain homozygotes and had to use another strategy (HET × HET) in subsequent generations to propagate the mutant. This phenomenon also exists in other *Orco* mutant insects, for example *Spodoptera littoralis* [71] and *M. sexta* [42]. There are two possible reasons for this phenomenon. Sperm activity might be impaired when *Orco* is knocked out in male moths. In mosquitoes and some mammalian species, some Ors and Orco are involved in the activation and (possibly) orientation of spermatozoa in male germ cells [72–74]. Another possible explanation is that *Orco*^−*/*−^ males could not perceive sex pheromones released by female moths. At the same time, we found that *Orco*^−*/*−^ females were able to mate with WT males in our oviposition test and healthy progeny were obtained (Fig. 2, Fig. 10). In contrast with these results, a previous study found that *Orco*^−*/*−^ females had significantly impaired fecundity (e.g., delayed oviposition and fewer eggs). In *Harpegnathos saltator* ants, *Orco*^−*/*−^ males had an equivalent mating ability to WT males [64]. An interesting question for future studies is how *Orco* affects the fecundity of the different sexes in these two insects.

Orco seems indispensable in detection of sex pheromone and plant odor in moth species. In *B.* *mori*, the males of *BmOrco* mutant displayed a significantly impaired mating selection behavior in response to the sex pheromone mixture of bombykol and bombykal (11:1), and the mutant larvae displayed defective selection for mulberry leaves and different concentrations of the volatile compound cis-jasmone [69]*.* In *S.* *littoralis*, Orco knockout caused defects in plant odor and sex pheromone olfactory detection in homozygous individuals [71]. In *H.* *armigera*, it has been showed that OR-mediated olfaction is involved in plant volatile detection by adults and larvae [54, 55]. The present study shows that the antennal electrophysiological responses of the *Orco*^−*/*−^ to sex pheromone components and plant volatile substances were abolished, the upwind flight behaviors of *Orco*^−*/*−^ males to sex pheromones were severely reduced, the oviposition selectivity of the *Orco*^−*/*−^ females to the host plant green pepper has completely disappeared, and the chemotaxis of larvae to the green pepper was also lost in the *Orco*^−*/*−^ larvae. However, in *M. sexta*, a specialist insect with a classical interaction with *Datura wrightii*, the oviposition behavior was maintained in *Orco*^−*/*−^ females, which is mainly determined by the carboxylic acids 3-methylvaleric acid and hexanoic acid from feces [42]. These acids are sensed through IR-mediated olfaction in *M. sexta* [75].

The heteromultimeric complex of Orco and Orx expressed in OSNs plays a crucial role in insect olfaction, but little is known about the effect of *Orco* knockout on the expression of Ors and other chemosensory-related proteins. In *Orco*^−*/*−^ adults of *B.* *mori*, the expression levels of two *PR*s (*Or1* and *Or3*), *PBP1*, *PBP2*, and *PBP3* were decreased significantly in silkworm [69]. RNA sequencing revealed that *Orco* knockout caused differential expression of Or genes in the antenna of honey bees, but the expression of other types of chemoreceptor genes was generally unaffected [76]. We used transcriptional profiling to detect expression changes of *Ors*,* Irs*,* Grs*,* TRPA1*,* PBPs*, and *GOBPs* in antennae induced by the loss of *Orco*. There was no significant effect on the expression levels of most olfaction-related genes, but the expression of some genes increased or decreased. For example, the expression level of *Or11* and *Or13* were decreased in *Orco*^−*/*−^ males, while that of *Or11* was increased in *Orco*^−*/*−^ females. Or13 as the receptor of the major pheromone component, Z11-16: Ald is specifically expressed in male antennae, while Or11 is unbiasedly expressed in male and female antennae [11, 51]. Up to now, the function of Or11 is unknown [77]. It was reported that the OSN expressing Or13 are colocalized with the OSN expressing Or11 in the A type sensilla of males [78, 79], which may explain why the expression level of both *OR11* and *OR13* were decreased in males. However, why does the opposite effects of *Orco* knockout on *OR11* expression in two sexes deserves further investigation. In addition, the trafficking and dendritic localization of tuning Ors were strikingly impaired in Orco *Or83b* mutants of *D. melanogaster* [25]. Whether the transportation, trafficking, and localization of these Ors from cell body to neuronal dendrites in *H. armigera Orco*^−*/*−^ adults are scrambled or not remains unclear.

As the primary olfactory center in the insect brain, the AL has drawn a lot of attention in insect olfaction studies [80, 81]. There are numerous glomeruli (functional units of olfactory information processing) in the AL and ORNs expressing the same Or will converge on the same glomeruli [9]. The results of previous studies on changes in AL morphology caused by defective *Orco* were inconsistent [64]. In *D. melanogaster*, when *Orco* was knocked out, the neuroanatomy of the AL remained unchanged [27, 82]; however, in ants with expanded Ors, the number of glomeruli was severely reduced in two dependent *Orco* mutant alleles [64, 83]. In lepidopteran insects, the AL structure is sexually dimorphic. The MGC is specialized for processing sex pheromones in the ALs of males [84]. In *M. sexta*, the activity of ORNs in the MGC region was lost, the glomeruli existed, and the volume of the MGC region was reduced after *Orco* was knocked out [42]. In this study, we found that the number and volume of glomeruli in the AL and MGC region were very similar between the WT and *Orco*^−*/*−^ adults (Fig. 6). These results indicate that the development of glomeruli is independent of the development of OSN in *H. armigera*, which is in accordance with findings for *Drosophila* [85]. In *Drosophila*, OR gene expression begins only after AL patterning, and Orco is just localized exclusively to dendrites and OSN cell bodies [27].

## Conclusions

In summary, we established an *Orco* mutant using the CRISPR/Cas9 system and investigated the function of *Orco* in *H. armigera* using transcriptomics, neuroanatomy, electrophysiological recordings, and behavioral tests. Severe olfactory defects were observed in *Orco*^−*/*−^ moths, demonstrating a crucial role of Orco (OR-dependent olfactory responses) in survival, mating, and the location of host plants. This study not only demonstrated the important multifaceted role of Orco in the *H. armigera* olfactory system, but also highlights potential avenues for the development of novel pest control strategies that combine knock-in or even gene drive.

## Methods

### Insects rearing

*H. armigera* was originally obtained from Zhengzhou, Henan province of China, and have been reared in Institute of Zoology, Chinese Academy of Sciences, Beijing, for successive generations. The climate chambers were maintained under a 16:8 h (light: dark) photoperiod, at 26 ± 1 °C and 55–65% relative humidity. As previously reported [11], the larvae of *H. armigera* were fed on artificial diet in glass tubes. Pupae were sexed and placed in cages for eclosion. Adults were fed on 10% honey water.

### CRISPRCas9 gene editing methods

The sequence of *HarmOrco* was downloaded in NCBI (https://www.ncbi.nlm.nih.gov) and confirmed by our PCR analysis. With the help of online tool CHOPCHOP (http://ch opchop.cbu.uib.no/), single-guide RNA (sgRNA) targeting the sequence in exon 3 of *HarmOrco* was designed. And sgRNA was synthesized according to the manufacturer’s protocol (GeneArtTM Precision gRNA Synthesis Kit, Invitrogen). Cas9 protein was purchased from the company (Thermo Fisher Scientific, Shanghai, China).

Embryo microinjection was performed according to a reported method [4] with minor modifications. Briefly, embryos used for microinjection were collected within 1–2 h old after laid. The newly collected embryos were washed thoroughly with 10% sodium chloride solution for 1 min, followed by 5 min distilled water three times. After these washes, embryos were dried on filter paper and arranged on a coverslip using double-sided tape. A mixture of sgRNA (200 ng/µL) and Cas9 protein (100 ng/µL) was co-injected into embryos. The microinjection was performed under a Zeiss microscope. After microinjection, embryos were covered with a little flour and thereafter reared in a climate chamber to develop. Newly hatched larvae were timely transferred to artificial diet.

The genomic DNA was extracted from the hind leg of each G0 adult using the *TransDirect*® Animal Tissue PCR Kit (Beijing, China) as template for PCR. The gene-specific primers (forward primer; 5′-CGTAAAAAGTTATTATCGAATGGCA-3′; reverse primer: 5′-AGAACGCAAGCTTGATGATA-3′) were designed by NCBI Primer-BLAST (http://www.ncbi.nlm.nih.gov/tools/primer-blast/). The PCR reaction consisted of 12.5 µL Premix Taq, 6.5 µL ddH_2_O, 2 µL DNA template, 2 µL forward primer, and 2 µL reverse primer. Reaction conditions were as follows: denaturation for 5 min at 98 °C and 35 cycles of denaturation at 98 °C for 10 s, annealing at 55–60 °C for 30 s, and extension at 72 °C for 40 s. PCR products encompassing the target *Orco* sequence were sequenced to determine genotypes. Each G0 mutated adult was mated with three wild type adults. G1 heterozygotes carrying the same mutation were self-crossed to obtain homozygous mutant (*Orco*^−/−^) in G2. Significantly, there were no progeny when self-crossing homozygous mutant (*Orco*^−/−^), so heterozygotes (*Orco*^+/−^) in each generation were retained for self-crossing, and the homozygous mutants (*Orco*^−/−^) in the next generation were screened for experiments.

### Comparisons of lifespan between the *Orco*^*−/−*^ and WT of *H. armigera*

There were totally four groups used for lifespan statistics experiments: WT females, WT males, mutant females, and mutant males. In each group, thirty adults that emerged on the same day were placed in a cage and fed on 10% honey water. The number of surviving individuals in each group was counted daily until all individuals died. Three biological repetitions were performed.

### Antennal transcriptome sequencing from the *Orco*^−/−^ and WT of *H. armigera*

Antennae from males and females of the *Orco*^−***/***−^ and WT of *H.* *armigera* were collected separately for RNA-seq. All the insects were reared in the same condition, and the unmated three-day-old adults were used. In each group, antennae were dissected from nearly 30 moths and quickly stored at 80 °C until RNA extraction. Three biological replicates were conducted in each group. Total RNA was firstly extracted according to a previous work [86], then evaluated with Agilent 2100 BioAnalyzer (Agilent, USA) and an ND-2000 spectrophotometer (Nanodrop, Wilmington, DE, USA). High-quality RNA was used for cDNA library construction. And these libraries were sequenced on an Illumina HiSeq2000 platform with the Nova-PE150 mode. Clean data were de novo assembled with software Trinity [87]. Blast nucleotide database was created from the assembled Trinity.fasta file and were queried by the protein sequence file of OR genes annotation repertoire of *H. armigera* [44]. The putative OR transcripts were obtained with BLAST, and expression abundances were calculated with the RSEM package [88]. Differentially expressed genes (DEGs) were screened by threshold (a log_2_ fold change > 1 and *p* value < 0.05) though software DESeq2 [89]. The GO function enrichment of DEGs was completed by Omicshare tool (https://www.omicshare.com/).

### Comparisons of antennal lobe between *Orco*^−/−^ and WT adults

The neuroanatomy of antennal lobes from homozygous mutant (*Orco*^−/−^) and wild type moths were examined following a protocol previously published [90]. Brains were firstly dissected in phosphate-buffered saline (PBS) and then fixed in 4% paraformaldehyde overnight at 4 °C. Glomeruli were marked using the antibody SYNORF1 (Developmental Studies Hybridoma Bank, IA, USA) antibody and visualized with Alexa Fluor 488 goat anti-mouse secondary antibody (Invitrogen). Photos were obtained with a Zeiss LSM710 Meta laser scanning microscope (Zeiss, Oberkochen, Germany). The software AMERA 6.0 (ZIB, Germany) was used for construct brain atlas. Four adults were observed in each group.

### Chemicals and electrophysiology analysis

According to our previous method [91], antennal EAG (electroantennograms) responses using antennae from 3- to 5-day-old unmated wild type and mutant adults (females and males) were conducted. A total of 27 odorants including four previously identified pheromone gland components (Z11-16:Ald, Z9-16:Ald, Z9-14:Ald, Z11-16:OH) [50, 92], two green leaf volatiles (hexanal, trans-2-hexenal), four terpenoids (geraniol, farnesene, citral, linalool), seven aliphatic compounds (nonanal, 6-methyl-5-hepten-2-one, 2-octanone, acetoin, allyl-isothiocyanate, acetophenone, and 1-octanol), seven aromatic compounds (methyl salicylate, benzyl acetate, methyl phenylacetate, methyl benzoate, eugenol, ethyl benzoate, and benzaldehyde), two acids (acetic acid, propionic acid), and one amine (spermine) (Additional file 4: Table S2). All chemicals were diluted with paraffin oil, and the concentrations were set up as µg/µL. Briefly, 10 µL of each chemical was added into a filter paper (0.2 cm × 1 cm) in a pasture tube, and 10 µL paraffin oil was used for negative control. Ten repeats were conducted for each odorant. A computer was coupled with an IDAC-2 amplifier and used for record data, and all EAG data were analyzed with software EAG2000 (Syntech, Hilversum, the Netherlands).

Single sensillum recordings (SSRs) were conducted only using 3- to 5-day-old, unmated males from homozygous mutant (*Orco*^−/−^) and wild type. Following the previously described methods in *H. armigera* [93], six previously identified pheromone gland components (Z11-16: Ald, Z9-16: Ald, Z9-14: Ald, Z11-16: OH, Z9-16: OH, Z11-16: Ac) [93] were detected in our experiments. Similar with EAG responses experiments, odorant cartridges were made by loading 10 µL of stimulus (10 µg/µL) onto a filter paper. Briefly, a moth was fixed in a 1 mL disposable Eppendorf pipette tip with a narrow end cut. The head and antenna of the moth were protruded from the narrow end, then immobilized by the dental wax. An electrolytically sharpened tungsten electrode was inserted into the base of a single sensillum in the antenna and used for record signals. Another tungsten electrode was inserted into a compound eye of the moth as a reference electrode. For odorant delivery, a flow of humidified and purified air continuously blew to the antenna (12.5 mL/s) from a 15-cm-long steel tube. When tested, a 200-ms air pulse was generated after a stimulus was added into the air stream using a stimulus flow controller (CS-55, Syntech, Hilversum, the Netherlands) and the air pulse was delivered with a stable flow rate of 10 mL/s. The recorded signals were displayed through the IDAC interface amplifer (IDAC-4, Syntech, Hilversum, the Netherlands) and further analyzed with the software Autospike, version 3.4 (Syntech, Hilversum, the Netherlands).

### Wind tunnel assay of male adults

A mixture of Z11-16: Ald and Z9-16: Ald at a ratio of 97:3 (the principal sex pheromone components in *H. armigera* [92]) was used in our wind tunnel assay. Different from above electrophysiology analysis, the binary sex pheromone components (Z11-16: Ald, Z9-16: Ald) were dissolved in hexane and the concentration was 0.1 µg/µL. In order to adapt experiment conditions (0.45 lx of red light, 22–25 °C, and 20–40% RH), each virgin male (4-day-old) was placed in a wind tunnel (size: 2.5 m × 1 m × 1 m (L × W × H)) for at least 30 min prior to the experiment.

When tested, 10 µL solution was added to the filter paper and the wind speed was at 0.5 m/s. One virgin male was transferred into a cylindrical mesh cage, and their behaviors were observed for 5 min. Five different behavioral responses were recorded: (1) Flight: male moth quit out of the cage and took off; (2) Upwind: male moth flew against the wind or shown a zigzag pursuing flight model; (3) Close: male moth flew close to the pheromone source less than 10 cm; (4) Landing: male moth contacted and landed on the filter paper; (5) Copulate: male moth showed a series of behaviors such as curling abdomen, stretching the hair-pencils from the abdominal cavity. Thirty repetitions were carried out for both homozygous mutant (*Orco*^−/−^) and wild type.

### Oviposition choice analysis of female adults

In order to detect whether oviposition preference of female moth was impaired in homozygous mutant (*Orco*^−/−^), a dual-choice assay was conducted following the method described [4]. At the beginning, newly emerged mutant females or wild type females were respectively mated with wild type males for 3 days (sex ratio = 1:3). Then 3 gravid females were transferred into a cylinder cage (size: diameter 24 cm, height 26 cm) to lay eggs. A piece of gauze covered on the top side of cage as an oviposition substrate and was equally divided into four sections. When scotophase begins, two 1.5-cm-diameter fresh green pepper fruit discs were placed on the opposite corners of cage, no fruit discs were placed on another two corners as control. To avoid the contact clues, a stainless net shelf was placed between gauze and green pepper fruit discs (Additional file 8: Figure S4). After 24 h, numbers of eggs were manually counted in different oviposition areas and a new piece of gauze was placed on the cage. This dual-choice assay was continued for 3 days, and the mean number of eggs was recoded. Five repetitions were conducted for both mutant females and wild type females. According to the formula ((*T* − *C*)/(*T* + *C*)) [4], preference index was calculated.

### Larval chemotaxis analysis

Larval chemotaxis experiments were performed according to the previously reported work in silkworm [39] with some modifications. Four 1.5-cm-diameter fresh green pepper fruit discs and four 1.5-cm-diameter filter papers were arranged at opposite ends in a closed box (30 cm × 20 cm × 10 cm). A single fifth-instar larva was starved for 2 h prior to the onset of the experiment and was placed in the middle of the box. A video camera was used to track the behavior trajectory of larva (Additional file 9: Figure S5). Once the larva touched the green pepper or filter papers, the video recording was stopped. Each larva was recorded and observed up for 30 min. Videos were analyzed with the software EthoVision XT (v.11.5, Noldus Information Technology). Eight biological replications were conducted for each genotype.

### Statistical analysis

Multiple *t*-test was used to analyze the below experiments (comparisons of lifespan between wild types and *Orco*^−*/*−^ adults, the comparisons of genes expression levels between wild types and *Orco*^−*/*−^ adults, comparisons of antennal lobe between wild types and *Orco*^−*/*−^ adults, SSR data, wind tunnel assay of male adults, oviposition choice analysis of female adults, and larval chemotaxis analysis) with *, *P* < 0.05; **, *P* < 0.01; ***, *P* < 0.001. ANOVA followed by Tukey’s test was used for the comparisons of antennal EAG responses between wild types and *Orco*^−*/*−^ adults. All statistical analyses were conducted by the software GraphPad Prism 7.

## Supplementary Information

**Additional file 1. Figure S1.** (A) Sequences of different mutations in G1 from G0 (germinal mutations occurred in these G0 individuals). The top sequence represented wild type. The target sequences were in bold and PAM sequences (CGG) was highlighted with red. Expected cut sites were marked by a red triangle. Among these mutations, deletions were indicated by dotted lines and insertions by red lower case letters. (B) Phenotypes of homozygous mutant (*Orco*^-/-^) and wild type adults.

**Additional file 2. Table S1.** The TPM value of main olfactory related genes in *Orco*^*-/-*^ and wild type.

**Additional file 3. Figure S2.** (A, B) TPM of Irs in females and males. (C, D, E, F) TPM of other olfactory related genes in females and males. Multiple *t* test was used with *, *P* < 0.05; **, *P* < 0.01; ***, *P* < 0.001 as significant differences. Error bars, mean ± SD.

**Additional file 4. Table S2.** Summary of the chemicals used in our experiments.

**Additional file 5. Figure S3.** Summary of the recorded sensilla types according to the response profiles [50] in wild type (n = 72) (A) and homozygous mutant males (n = 70) (B). Type A sensilla only responded to Z11-16: Ald, while type B sensilla responded to Z9-14: Ald. Sensilla responding to Z9-16: Ald and Z9-14: Ald were classified as type C. The remaining sensilla were considered as “others” in pie chart.

**Additional file 6. Video S1.** A typical wind tunnel trajectory of a wild type male.

**Additional file 7. Video S2.** A typical wind tunnel trajectory of an *Orco*^*-/-*^ male.

**Additional file 8. Figure S4.** The set-up of oviposition choice tests (A, B) and the spread of eggs laid by mated females: (C) wild type females, (D) *Orco*^*-/-*^ females.

**Additional file 9. Figure S5.** Setup for behavioral tracing of a larva in a dual-choice arena system.

## Data Availability

All data generated or analyzed during this study are included in this published article, its supplementary information files and publicly available repositories. Sequencing data generated in this study has been uploaded to SRA database of NCBI under PRJNA813394.

## Abbreviations

Orco: Odorant receptor co-receptor
Ors: Odorant receptors
Irs: Ionotropic receptors
Grs: Gustatory receptors
CRISPR: Clustered regularly interspaced short palindromic repeats
Cas9: CRISPR-associated protein 9
*H. armigera*: *Helicoverpa armigera*
AL: Antennal lobe
MGC: Macroglomerular complex
Z11-16:Ald: (Z)-11-hexadecenal
Z9-14:Ald: (Z)-9-tetradecenal
Z9-16:Ald: (Z)-9-hexadecenal
Z9-16:OH: (Z)-9-hexadecenol
Z11-16:OH: (Z)-11-hexadecenol
Z11-16:Ac: (Z)-11-hexadecenyl acetate
EAG: Electroantennogram
SSR: Single sensillum recording
ORNs: Odorant receptor neurons
GPCRs: G-protein coupled receptors
PRs: Pheromone receptors
sgRNA: Single-guide RNA
WT: Wild type
DEGs: Differentially expressed genes
GO: Gene ontology
TPM: Transcripts per million
TRPA1: Transient receptor potential cation channel subfamily A member 1
PBP: Pheromone-binding protein
GOBP: General odorant-binding protein
ANOVA: Analysis of variance
