# Supplementary figures and images for: Mutagenesis of the odorant receptor co-receptor (Orco) reveals severe olfactory defects in the crop pest moth Helicoverpa armigera
Source: BMC Biol. 2022 Sep 30;20:214. doi: 10.1186/s12915-022-01411-2 (PMC9524114; doi:10.1186/s12915-022-01411-2)

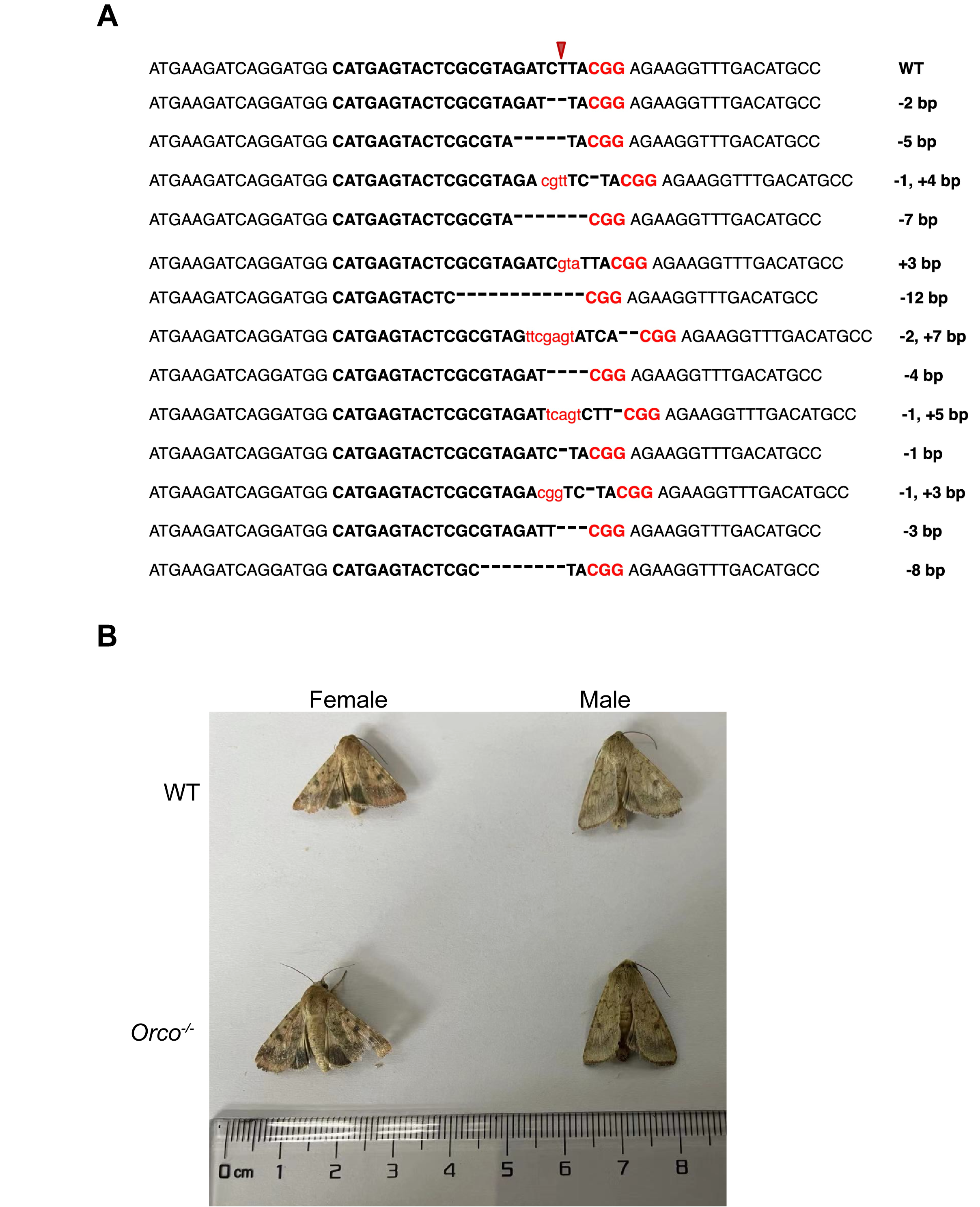

Supplement: Supplementary file 1 — Additional file 1. Figure S1. (A) Sequences of different mutations in G1 from G0 (germinal mutations occurred in these G0 individuals). The top sequence represented wild type. The target sequences were in bold and PAM sequences (CGG) was highlighted with red. Expected cut sites were marked by a red triangle. Among these mutations, deletions were indicated by dotted lines and insertions by red lower case letters. (B) Phenotypes of homozygous mutant (Orco-/-) and wild type adults. [file 12915_2022_1411_MOESM1_ESM.tif]

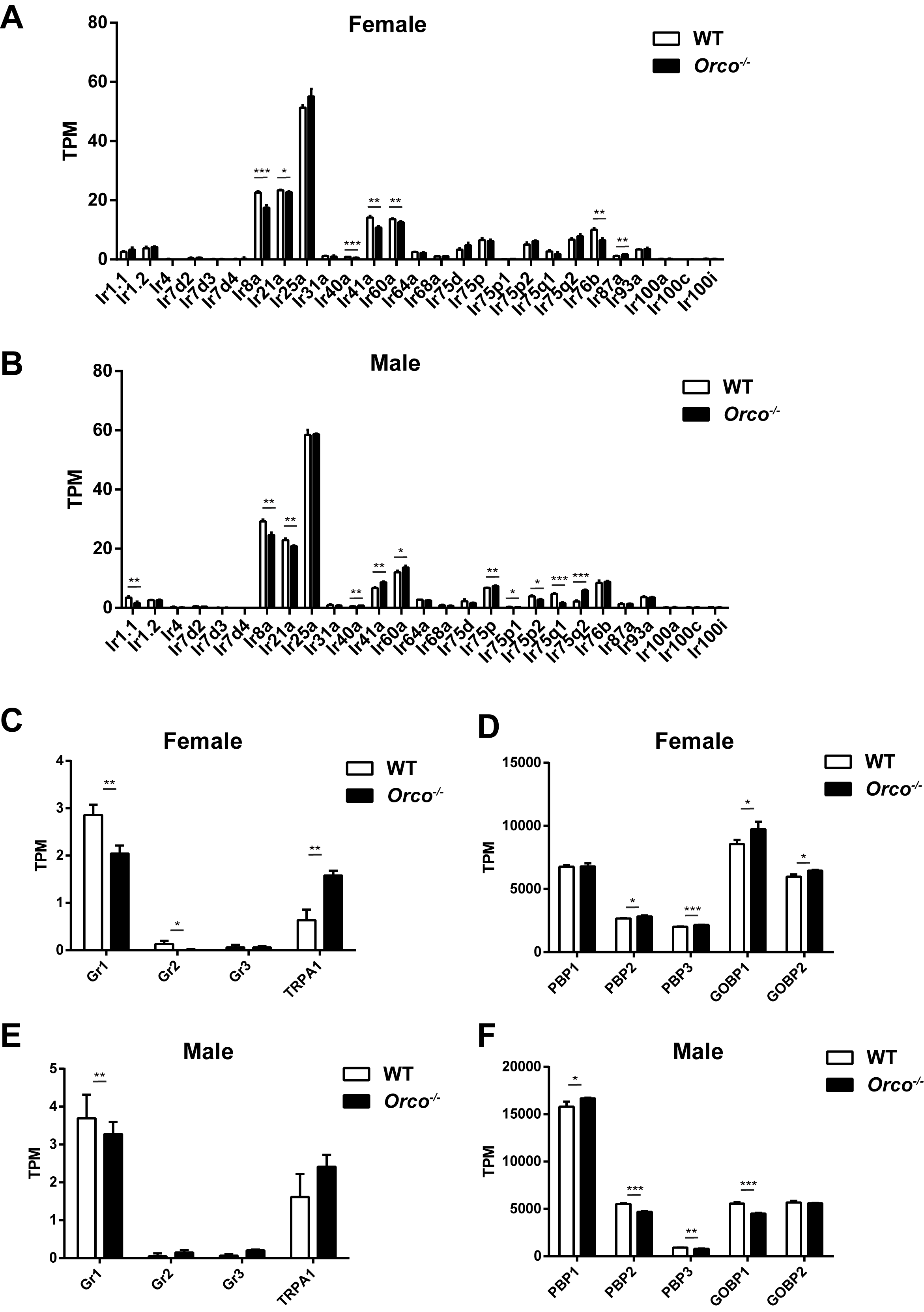

Supplement: Supplementary file 3 — Additional file 3. Figure S2. (A, B) TPM of Irs in females and males. (C, D, E, F) TPM of other olfactory related genes in females and males. Multiple t test was used with *, P < 0.05; **, P < 0.01; ***, P < 0.001 as significant differences. Error bars, mean ± SD. [file 12915_2022_1411_MOESM3_ESM.tif]

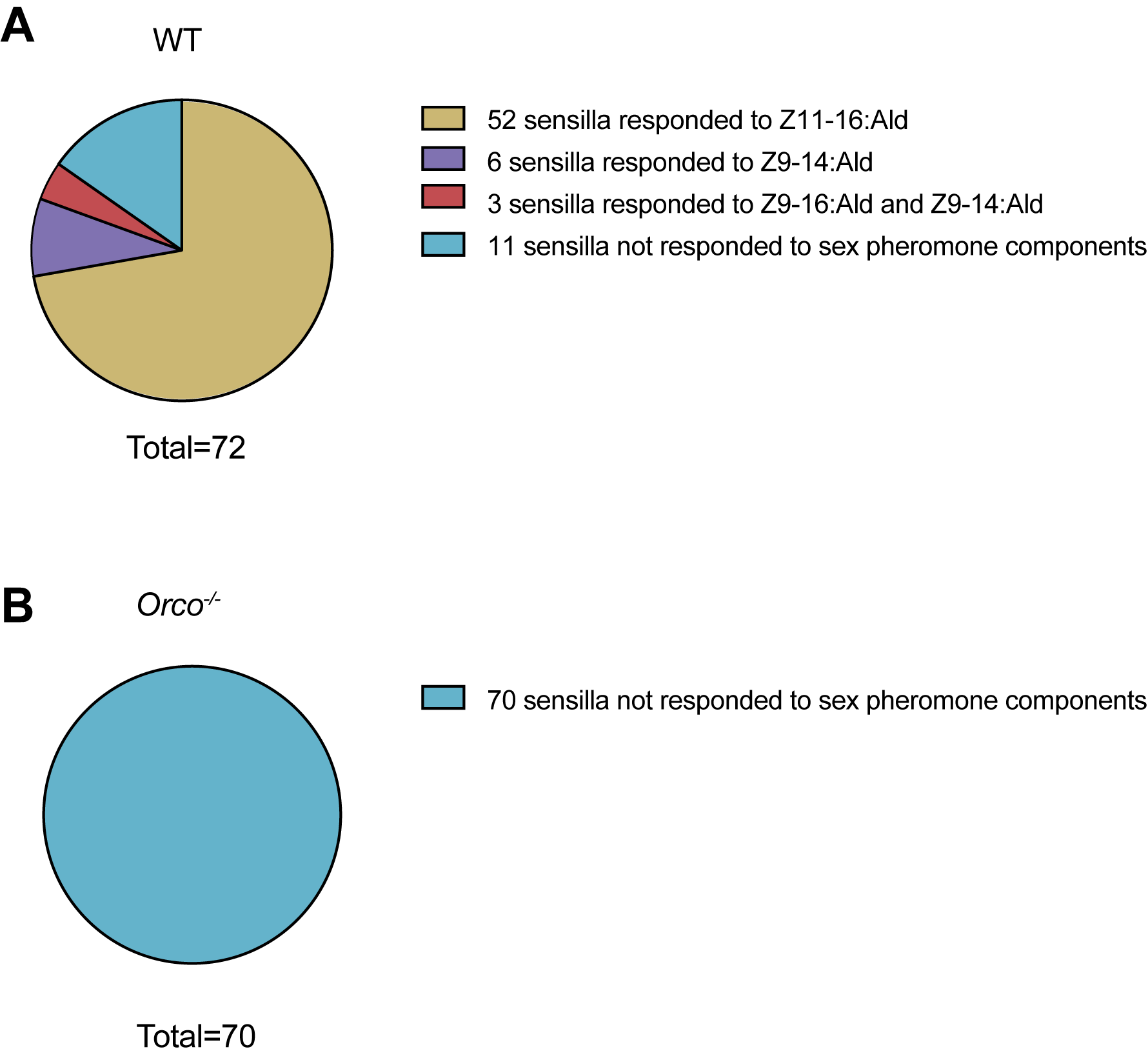

Supplement: Supplementary file 5 — Additional file 5. Figure S3. Summary of the recorded sensilla types according to the response profiles [50] in wild type (n = 72) (A) and homozygous mutant males (n = 70) (B). Type A sensilla only responded to Z11-16: Ald, while type B sensilla responded to Z9-14: Ald. Sensilla responding to Z9-16: Ald and Z9-14: Ald were classified as type C. The remaining sensilla were considered as “others” in pie chart. [file 12915_2022_1411_MOESM5_ESM.tif]

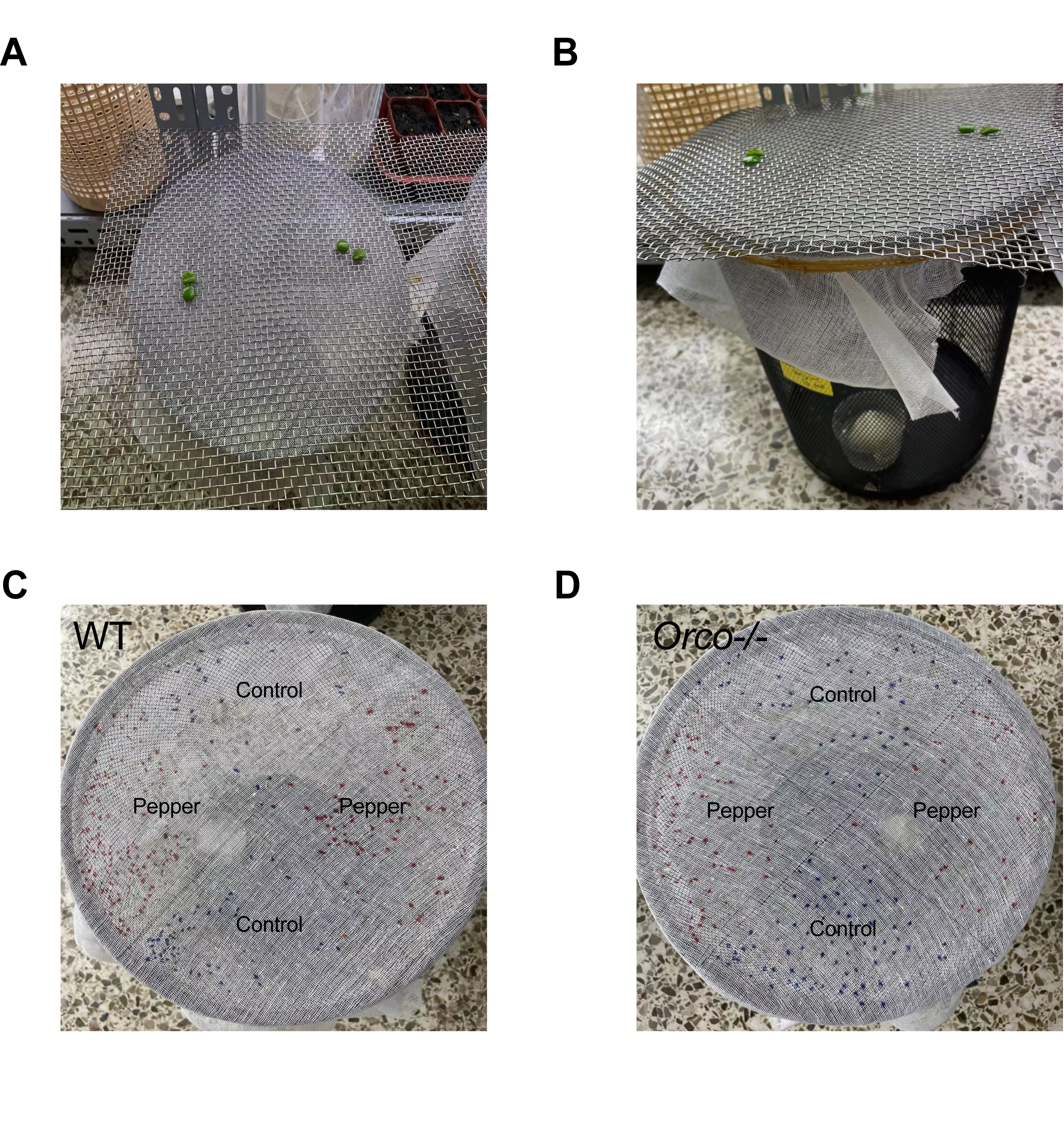

Supplement: Supplementary file 8 — Additional file 8. Figure S4. The set-up of oviposition choice tests (A, B) and the spread of eggs laid by mated females: (C) wild type females, (D) Orco-/- females. [file 12915_2022_1411_MOESM8_ESM.tif]

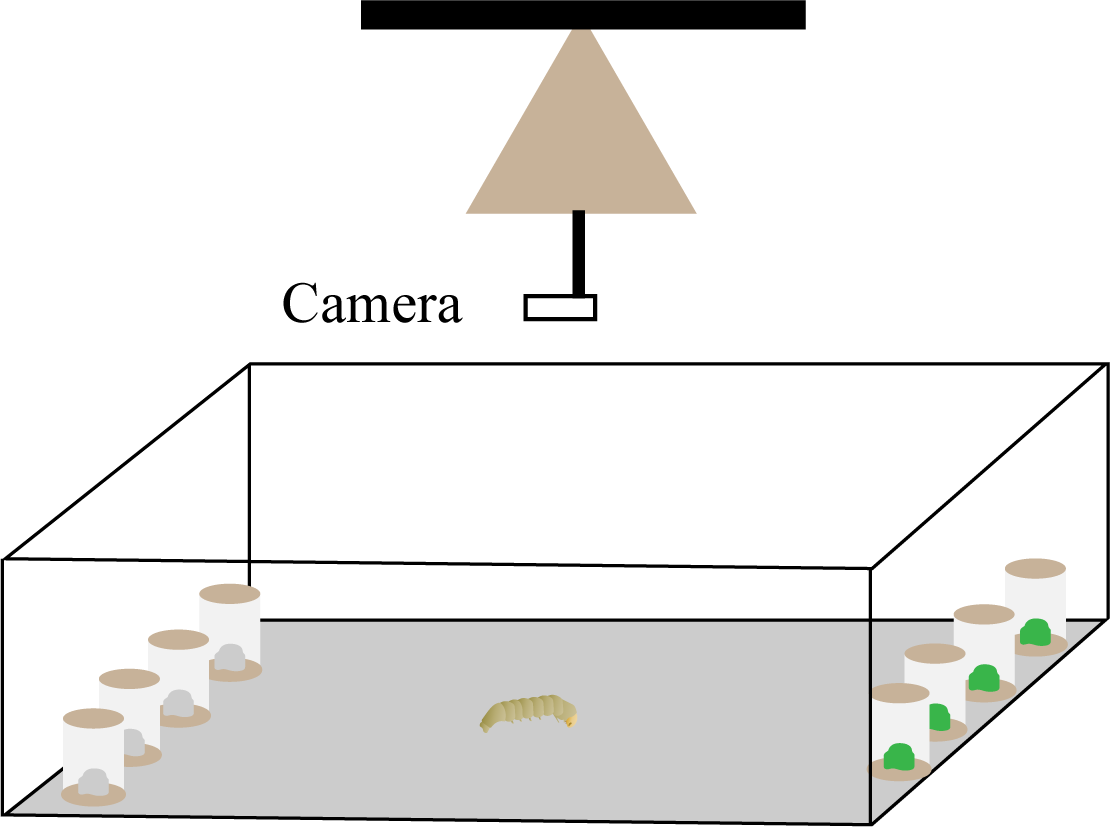

Supplement: Supplementary file 9 — Additional file 9. Figure S5. Setup for behavioral tracing of a larva in a dual-choice arena system. [file 12915_2022_1411_MOESM9_ESM.tif]
